# Value of liver iron concentration in healthy volunteers assessed by MRI

**DOI:** 10.1038/s41598-020-74968-z

**Published:** 2020-10-21

**Authors:** Marzanna Obrzut, Vitaliy Atamaniuk, Kevin J. Glaser, Jun Chen, Richard L. Ehman, Bogdan Obrzut, Marian Cholewa, Krzysztof Gutkowski

**Affiliations:** 1 Department of Biophysics, College of Natural Sciences, Institute of Physics, University of Rzeszow, Rzeszow, Poland; 2 Department of Radiology, Mayo Clinic, Rochester, MN USA; 3 Department of Obstetrics and Gynecology, Institute of Medical Sciences, Medical College, University of Rzeszow, Rzeszow, Poland; 4 Department of Gastroenterology and Hepatology with Internal Disease Unit, Teaching Hospital No. 1, Rzeszow, Poland

**Keywords:** Liver, Magnetic resonance imaging

## Abstract

Iron overload is a relatively common clinical condition resulting from disorders such as hereditary hemochromatosis, thalassemia, sickle cell disease, and myelodysplasia that can lead to progressive fibrosis and eventually cirrhosis of the liver. Therefore, it is essential to recognize the disease process at the earliest stage. Liver biopsy is the reference test for the assessment of liver fibrosis. It also allows for quantifying liver iron concentration (LIC) in patients. However, this is an invasive method with significant limitations and possible risks. Magnetic resonance imaging (MRI) and evaluation of the R2* relaxation rate can be an alternative to biopsy for assessing LIC. However, it causes a need for accurate R2* data corresponding to standard value for further comparison with examined patients. This study aimed to assess the normative values of liver R2* in healthy individuals. A total of 100 volunteers that met established criteria were enrolled in the study: 36 (36%) men and 64 (64%) women. The mean age was 22.9 years (range 20 to 32 years). R2* was estimated by an MRI exam with a 1.5 T clinical magnetic resonance scanner. Images for measuring the LIC and liver fat concentration were obtained using the IDEAL-IQ technique for liver imaging. The Mean (SD) liver R2* was 28.34 (2.25) s^−1^ (95% CI, 27.78–28.90, range 23.67–33.00 s^−1^) in females, 29.57 (3.20) s^−1^ (95% CI, 28.49–30.66, range 23.93–37.77 s^−1^) in males, and 28.72 (2.69) s^−1^ (range 23.67–37.77 s^−1^) in the whole group. R2* value in this particular population with a high proportion of young women did not exceed 38 s^−1^. In the absence of fibrosis or steatosis, liver stiffness and fat fraction did not show any relationship with R2*.

## Introduction

Iron overload is a relatively common clinical condition, resulting from disorders such as hereditary hemochromatosis, thalassemia, sickle cell disease, and myelodysplasia^1^. Iron accumulation can result from excessive iron supply or absorption. The most common cause of excessive iron supply is secondary to red blood cell (RBC) transfusions used to treat chronic anemia (e.g., thalassemia, sickle cell disease, inherited bone marrow failure syndrome, and myelodysplastic syndrome). A less common cause is the use of supplements or therapeutic infusions rich in iron, such as hemin used to treat some types of porphyria.

The most common cause of increased iron absorption in adults is congenital hemochromatosis caused by gene mutation, inefficient erythropoiesis in the course of thalassemia, sideroblastic and other hereditary anemias, and liver diseases (in particular, acute liver disease (ALD) and chronic diseases like nonalcoholic fatty liver disease (NAFLD) and chronic viral hepatitis). Usually, patients have more than one reason for iron overload. The mechanism for iron absorption in the liver is not fully understood and is most likely associated with a decrease in hepcidin production in the diseased liver^2^.

Irrespective of the causes of iron overload, the liver is the organ that is most affected by iron overload. In the case of increased intestinal iron absorption due to the accumulation and damage of hepatocytes, this can lead to progressive fibrosis and eventually cirrhosis of the liver, resulting in portal hypertension, liver failure, and hepatocellular carcinoma^3,4^. Therefore, it is extremely important to recognize the disease process in as early a stage as possible. Liver biopsy is considered to be the definitive test for the assessment of liver fibrosis. In the setting of iron overload, a biopsy can be used to quantify liver iron concentration (LIC)^5^. However, this is an invasive method with substantial sampling error. A significant limitation of this diagnostic method is the fact, that the distribution of iron in the liver is inherently very heterogeneous^6^. Besides, this method carries a risk of complications, such as postinterventional hemorrhage and bleeding^7,8^.

Due to the known systematic and accidental errors, costs, and invasiveness, a biopsy cannot be considered a gold standard for LIC assessment and should not be used for diagnostics except for disease processes requiring histological assessment of the organ to establish a diagnosis and correct treatment^6^.

Magnetic resonance imaging (MRI) techniques have been advocated as an alternative to biopsy for assessing LIC. One approach is to measure the R2* relaxation rate of liver tissue by using a multipoint Dixon sequence such as IDEAL-IQ^9^. Studies have shown that R2* increases systematically with hepatic iron concentration^10^. This parameter has a high correlation with biopsy measurements and could be a reliable, noninvasive method for measuring LIC^10^. For MRI to be used for the rapid routine assessment of LIC, it is necessary to establish ranges of R2* values for the healthy population. To our knowledge, there is little data in the literature assessing the liver iron level in healthy adult volunteers^11^. Published studies have qualified the absence of liver disease in cohorts by the participant's declaration of the absence of liver disease without the confirmation of this fact, even using basic laboratory tests. There is a need to obtain objective R2*data based on representative groups of healthy subjects who undergo more tests to confirm their health status, especially to confirm their lack of liver inflammation and fibrosis.

## Objectives

To assess the normative values of liver R2* measured by the IDEAL-IQ MRI sequence in healthy volunteers.

### Study enrollment

115 subjects were primarily enrolled in the study. The inclusion criteria were as follows: no contraindications to MRI, no history of liver disease, no risk factors for chronic liver disease, negative family history of chronic liver disease, normal liver enzyme levels, normal ferritin (13–150 ng/ml for women, 30–400 ng/ml for men) and iron values (37–170 mcg/dl for women, 49–181 mcg/dl for men), no drug or medication use of any kind and absence of liver fibrosis or steatosis on MRI. As exclusion criteria were used following values of parameters: liver stiffness < 2.9 kPa and fat fraction < 5%^12^. All subjects declared they consumed less than 30 g of alcohol per day. The participants were not required to change their normal diet to be included in this study but required 6 h of fasting before the MRI scans. The research was carried out in the magnetic resonance laboratory of the Center for Medical and Natural Sciences Research and Innovation of the University of Rzeszow, Poland in the period from December 2018 until July 2019.

### Study protocol

Subjects were examined on a 1.5-T whole-body OPTIMA MR360 Advance MR scanner (GE Healthcare, Milwaukee, Wisconsin, United States) with software version SV23. This device is equipped with a gradient system with a maximum amplitude of 33 mT/m and a 120 mT/m/ms slew rate. For liver MRI and MRE examinations, all patients underwent the scan in the feet-first supine position with an 8-channel torso phased-array coil around the abdomen. As part of the MRE setup, a drum-like passive liver driver was secured to the right side of the chest wall with its center at the superior-inferior (SI) level of the xiphoid process. There was a 29-foot long PVC tube (3/4-inch diameter) connecting the passive driver to an active driver (the source of the vibrations used during MRE acquisitions) located in the adjacent equipment room.

Images for measuring the LIC and liver fat concentration were obtained using the IDEAL-IQ technique for liver imaging (GE Healthcare, Waukesha, WI, USA) and was performed using a gradient-echo multi-echo MR sequence with the following imaging parameters: FOV = 39 cm, TR = 12.4 ms, acquisition matrix = 160 × 160, slice thickness = 10 mm, slice number = 28. Six echo times were collected and R2* and fat-fraction images were generated automatically on the scanner. The acquisition was performed in a single breath-hold of 17 s.

Liver stiffness images were obtained using the MR-Touch MRE technique (GE Healthcare, Waukesha, WI, USA) using a 2D gradient-echo pulse sequence with a 60-Hz motion frequency. The imaging parameters were as follows: FOV = 40 cm, TR = 33.3 ms, TE = 20.5 ms, acquisition matrix = 224 × 64, parallel imaging factor of 2, reconstructed matrix size = 256 × 256, slice thickness = 10 mm, flip angle = 30°, number of time offsets = 4, time of acquisition = 1 min 8 s. Nine slices were obtained through the liver in three or four breath holds, and the subjects held their breath at the end of expiration. Images of the liver stiffness (elastograms) were generated automatically on the scanner and were transferred offline for additional analysis and measurements.

### Image analysis

To measure liver R2* and fat concentration, four irregular polygon regions of interest (ROIs) were defined in the liver on four consecutive R2* images and were applied to the fat content images as well. ROIs were drawn to be as large as possible while excluding large vessels and the central biliary tree. Besides, the position of the ROIs was chosen in areas far from the organ boundaries. Liver R2* was calculated as the mean of the four R2* values and was used to estimate LIC according to Wood formula ([Fe] mg/g = R2* × 0.0254 + 0.202)^6^. Since not all our patients had saved individual eight echoes in the clinic scans for calculating the offline classic R2* results by using the magnitude-based monoexponential model^6^, we used the scanner-generated the complex-based IDEAL-IQ R2* results. A newly published study^13^ found the classic R2* and IDEAL-IQ R2* were significantly correlated to each other (r = 0.9231, *p* < 0.0001), and the patient R2* range covered our patients R2*.

Liver stiffness was measured by placing a total of four irregular polygon ROIs in the right hepatic lobe on the elastograms, with large vessels and regions of low confidence excluded. Liver stiffness was calculated as the mean of the mean liver stiffness values obtained from each of the four middle slices, weighted by the ROI area.

### Statistical analysis

Liver stiffness and liver R2* values were presented as mean (SD). To assess the correlation of liver R2* with liver stiffness, liver fat fraction, and ferritin level, the Pearson correlation test was performed. The Mann–Whitney U test was used to identify differences between male and female volunteers. Statistical significance was assumed at a *p* value of less than 0.05. MedCalc software (version 18.9.1; Mariakerke, Belgium) was used to perform the statistical analysis.

### Ethics

The study protocol was approved by the Ethics Committee of the Medical Department of Rzeszow University (Resolution No. 8/10/2016) and conformed to the ethical guidelines of the 1975 Declaration of Helsinki (6th revision, 2008). After receiving an explanation of the study, each subject provided written informed consent to enroll in the study.

## Results

Of the 115 volunteers, 15 were excluded because of elevated (abnormal) values of aspartate aminotransferase (AspAT) and/or alanine aminotransferase (AlAT). The remaining 100 were considered eligible and were included in the study. All MRE exams were successfully completed. None of the volunteers reported any discomfort during the procedure. Of these 100 subjects, 36 (36%) were male and 64 (64%) were female and the mean age was 22.9 years (range 20 to 32 years). The mean BMI for whole group was 21.33 kg/m^2^ (SD 2.19 kg/m^2^, range: 16.85–25.76 kg/m^2^. For female volunteers the mean BMI was 20.64 kg/m^2^ (SD: 1.96 kg/m^2^, range: 16.85–25.59 kg/m^2^. For men, the mean BMI was 22.56 kg/m^2^ (SD: 2.05 kg/m^2^, range: 17.96–25.76 kg/m^2^.

**Figure 1:**
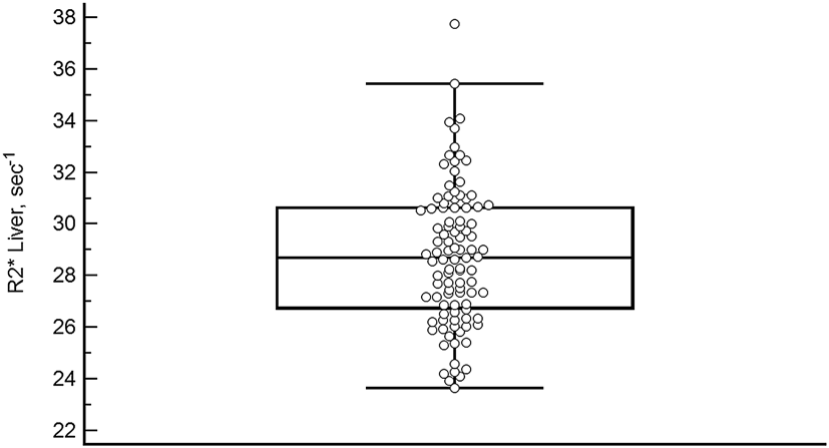
Liver R2* values in normal volunteers. Box-and-whisker plot graph showing the distribution of the liver R2* values in the 100 normal healthy volunteers. Dots represent the value of each volunteer.

To measure liver R2*, the average size of the ROIs was 5 to 7 cm^2^ for the liver. The mean (SD) liver R2* for the population was 28.72 (2.69) s^−1^ (95% CI, 28.26–29.32, range 23.67–37.77 s^−1^) (Fig. 1). The hepatic iron load was assessed using the Wood formula ([Fe] mg/g = R2* × 0.0254 + 0.202). This equates to a mean LIC of 0.93 mg/g (range 0.80–1.16 mg/g)^6^.

**Figure 2:**
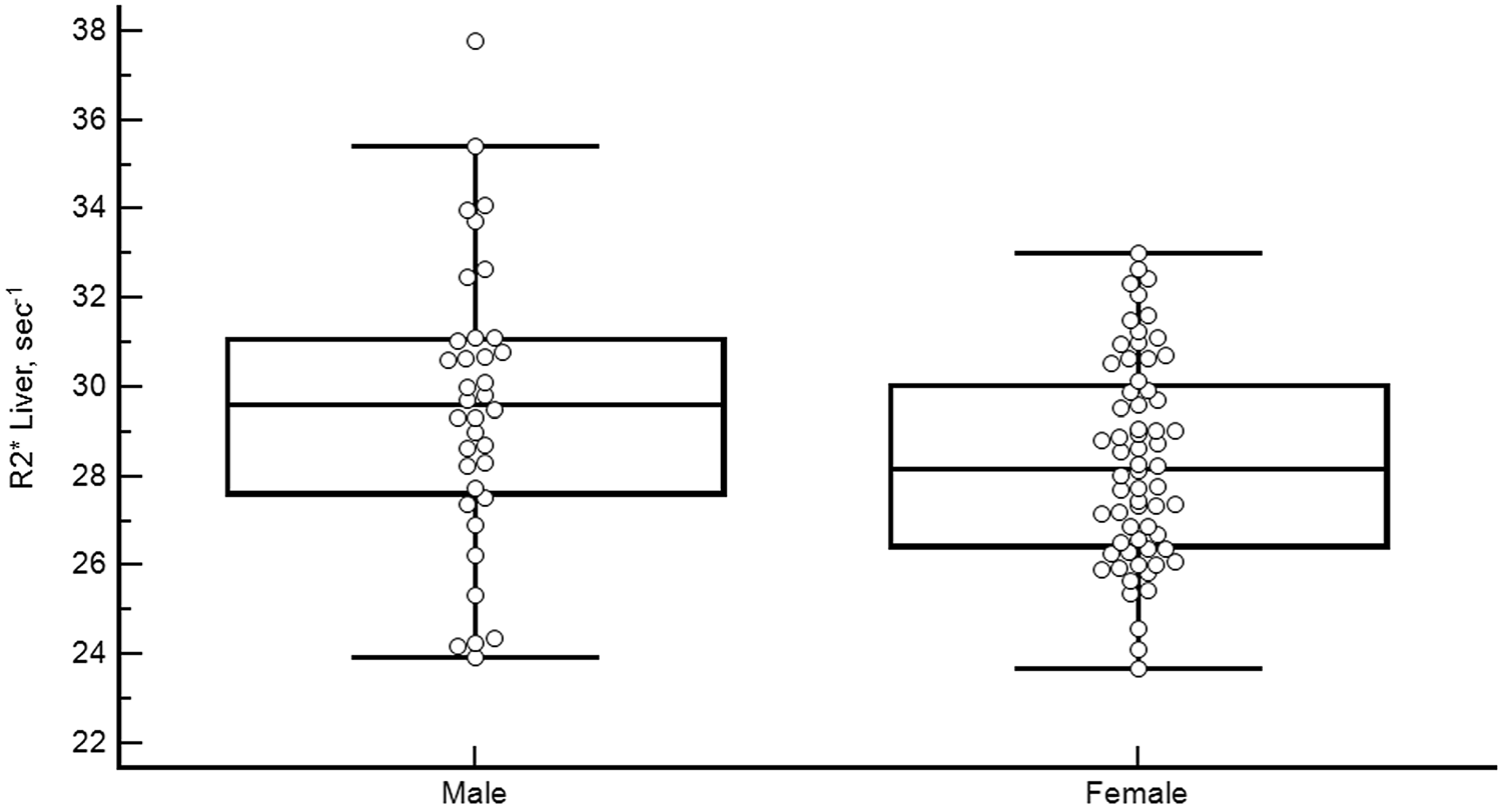
Liver R2* values separated by sex. Boxplot graph showing the distribution of liver R2* values in the female and male volunteers.

Mean (SD) liver R2* for the females was 28.34 (2.25) s^−1^ (95% CI, 27.78–28.90, range 23.67–33.00 s^−1^). For males, the mean was 29.57 (3.20) s^−1^ (95% CI, 28.49–30.66, range 23.93–37.77 s^−1^) (Fig. 2). This difference was statistically significant (*p* = 0.04).

**Figure 3:**
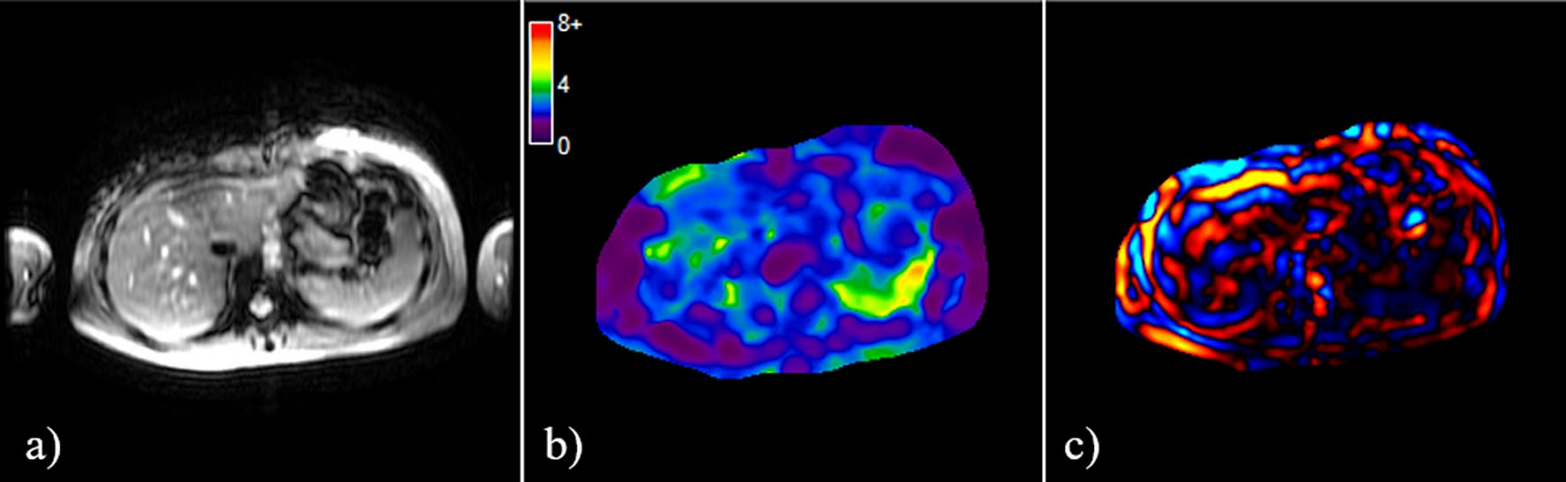
An example of MRE scan showing (**a**) magnitude, (**b**) elastogram, and (**c**) wave images.

Figure 3 shows an example of an MRE scan. The liver stiffness in healthy volunteers ranged from 1.84 to 2.82 kPa, with a mean (SD) of 2.30 (0.23) kPa (95% CI, 2.25–2.34). Mean (SD) liver stiffness for females was 2.31 (0.23) kPa (95% CI, 2.26–2.37, range 1.84–2.82 kPa) and for males was 2.27 (0.23) kPa (95% CI, 2.19–2.35, range 1.92–2.79 kPa). These values did not differ significantly (*p* = 0.35). There was no correlation between the liver stiffness and gender (*p* = 0.19). There was also no correlation between liver R2* and liver stiffness (r = − 0.04, *p* = 0.68) (Fig. 3).

Liver fat fraction ranged from 1.40 to 4.90%. Mean value (SD) was 2.51 (0.75)% (95% CI, 2.36–2.66%). For males, the mean was 2.69 (0.88) % (95% CI, 2.39–2.99%; range 1.70–4.90%). For females, the mean was 2.41 (0.66) % (95% CI, 2.25–2.58%; range 1.40–4.87%). There was no correlation between the liver fat fraction and gender (*p* = 0.19). Liver R2* did not correlate with liver fat fraction (r = 0.13, *p* = 0.20).

Ferritin level ranged from 3.74 to 260.10 ng/ml. Mean value (SD) was 53.29 (52.15) ng/ml (95% CI, 42.94–63.64 ng/ml). For males, the mean was 93.31 (64.56) ng/ml (95% CI, 71.46–115.16 ng/ml; range 6.22–260.10 ng/ml). For females, the mean was 30.78 (22.98) ng/ml (95% CI, 25.04–36.52 ng/ml; range 3.74–113.1 ng/ml). This difference was statistically significant (*p* < 0.0001).

**Figure 4:**
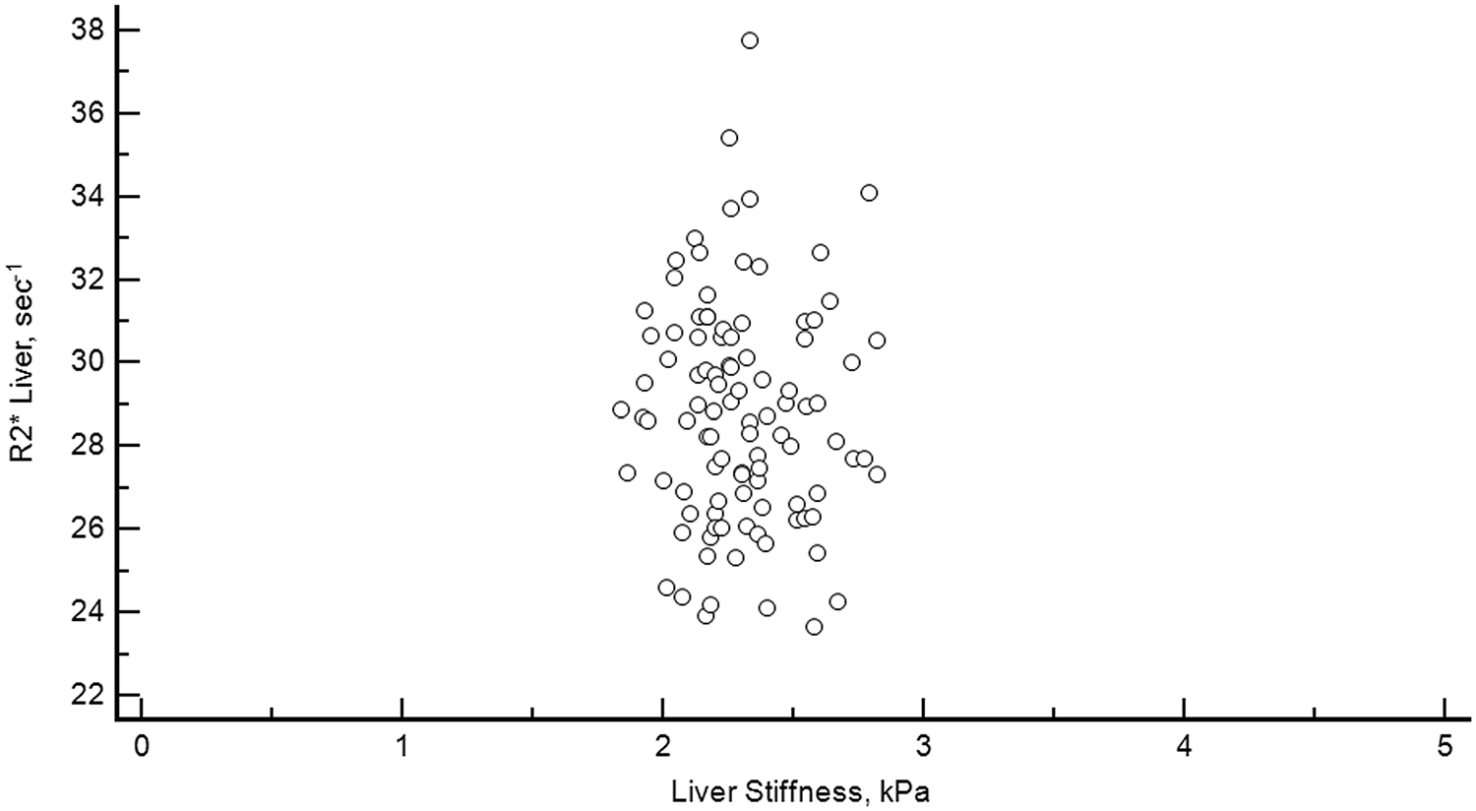
Relationship between liver stiffness and R2*. Scatterplot diagram showing the relation between liver stiffness and liver R2* in the 100 healthy volunteers.

**Figure 5:**
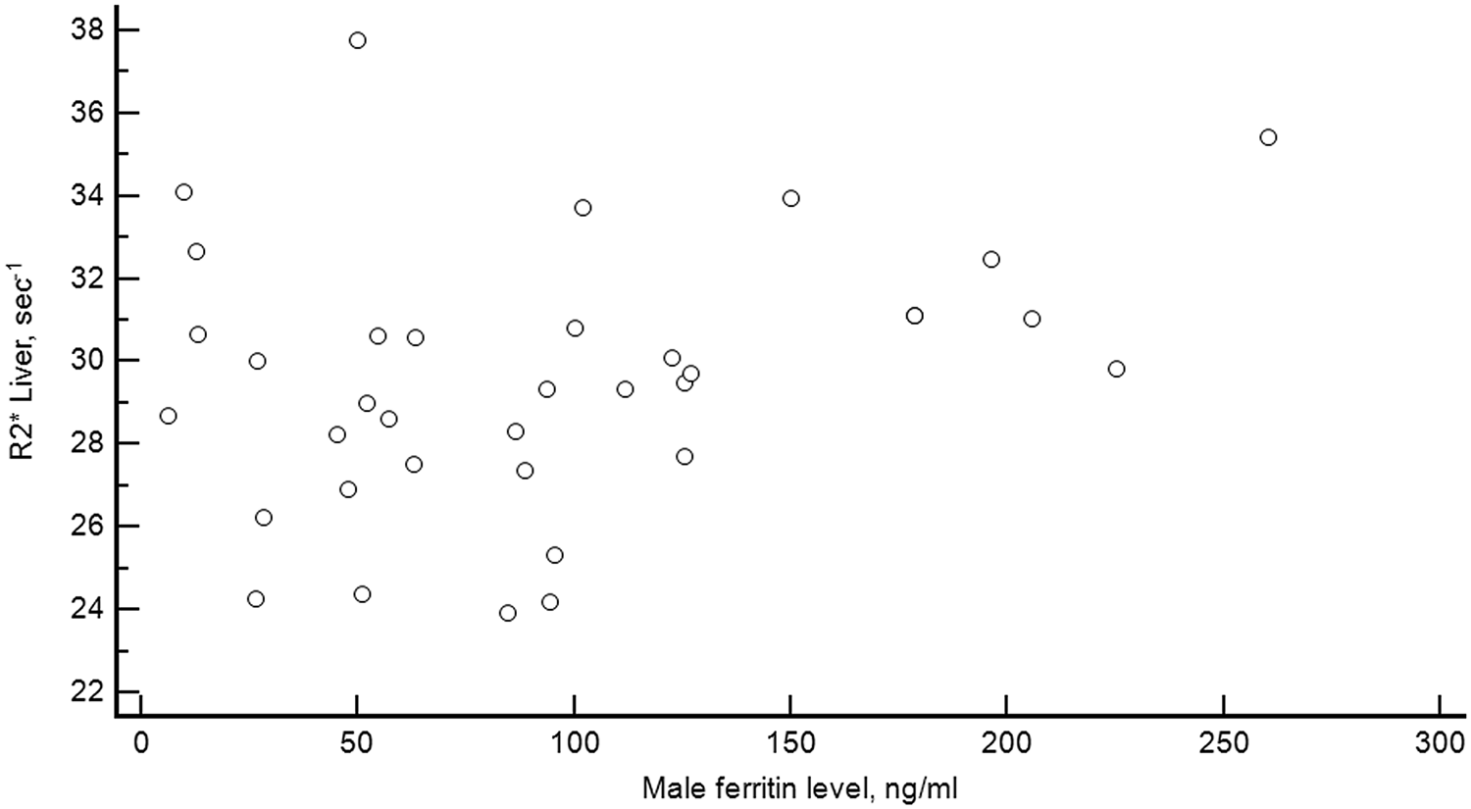
Relationship between ferritin level and liver R2* for males. Scatterplot diagram showing the relation between ferritin level and liver R2* in the 36 healthy male volunteers.

There was no correlation between liver R2* and ferritin level neither for males: r = 0.28, *p* = 0.096 (Fig. 4), nor for females: r = − 0.01, *p* = 0.91 (Fig. 5).

## Discussion

The normal iron content in the human body is 40 mg/kg in women by menopause and 50 mg/kg in men^14,15^. About 80% of total iron in the human body occurs in the red blood cells and in proteins other than hemoglobin. The remaining 20% is largely stored in the ferritin and hemosiderin^16,17^. Ferritin acts as a deposit storing excess iron and occurs primarily in the liver, spleen, and bone marrow^17^.

A smaller amount of iron is associated with transferrin in plasma^18^. In women, the amount of stored iron is lower and additionally depends on the severity of menstruation, pregnancy, lactation as well as the amount of iron supplements taken. Premenopausal women lose iron due to menstruation at a rate of about 0.5–1.0 mg/day.

There are no physiological mechanisms to eliminate iron from the body when there is excess^2^. If the supply of iron from the various sources is too high, then systemic iron overload occurs in the body. Initially, ferritin and hemosiderin (which is a partially denatured form of ferritin) store the excess iron. If the iron-binding capacity of the substances is exceeded, free iron is deposited in the cells of other organs.

Even if the level of ferritin in the blood can be used as a rough indicator of liver iron overload in a highly overloaded population^19^, it is considered that it does not allow the diagnosis of liver iron overload and should not be used solely for clinical decision making^20–22^.

In the past 20 more years, many MRI methods have been developed to measure hepatic iron concentration, including SIR methods at 0.5 T^23^, 1.5 T^24,25^, and 3 T^26^, R2 calculation at 1.5 T^1^ and R2* calculations at 1.5 T^6,21,27–29^ and at 3 T^30^.

Due to whole body iron metabolism^31^, MRI is the most practical and accurate method to monitor the concentration of iron in various human organs. The literature describes attempts to assess iron content in the body using R2* or T2*magnetization relaxation values (R2* = 1000/T2*). The majority of the studies showed a good correlation between iron concentration in the liver and T2 or T2* in patients with iron-overload diseases in the course of congenital hemochromatosis, thalassemia, and sickle cell disease^1,6,19,20,23,25,32–37^.

The MRI measurements hepatic R2* or T2* is a fast, widely available, and relatively inexpensive technique that some institutions are willing to use as an alternative to liver biopsies. However, for studies of T2*/R2* in healthy individuals, the literatures only reported a small number of participants^6,35^. Maris et al. measured the T2* value for the liver in 21 healthy volunteers^35^. In the Wood study, for comparative purposes, data were obtained in a 13-person control group^6^. The only more notable work assessing the levels of T2* in the liver and spleen in healthy volunteers and comparing these values with the level of ferritin in blood^11^ is of limited value due to the non-objective criterion of qualification: only a participant health declaration without basic corroborating laboratory tests.

The results from our study fill this existing gap in the literature. In our work, we included healthy volunteers with no history of liver disease and negative blood tests that excluded inflammatory processes in the body. The absence of fibrosis processes in the liver was additionally confirmed by MR elastography exam. The stiffness of the liver was in the range of 1.84–2.82 kPa with an average of 2.30 (0.23) kPa, which was within the normal range^38^.

In our study, the average value of R2* was 28.751 s^−1^ (T2* = 34.78 ms). For comparison, Anderson gives a T2* value of 33 ± 7 ms for the liver and 56 ± 22 ms for the spleen^27^. Maris et al. reported the average T2* for the liver as 24.2 ± 3.0 ms in 21 healthy volunteers^35^. The recommended upper limit of LIC is < 2 mg Fe/g dry liver tissue^39^. Another study by Nuttall et al., based on a series of 141 liver biopsies, suggests reference limit 1.8 mg Fe/g dry liver tissue in healthy adults^40^. The LIC increases with age, an average of 14 mcg Fe/g per year^40^. As expected, the LIC values in women are lower than in men^41^. The above-mentioned LIC value of 2 mg/g is equivalent to the R2* value of about 70 s^−1^ ^29^. The R2* value measured in our healthy volunteers corresponds well with the proposition of classification of iron overload severity offered by Henninger et al.^39^. In the 13 healthy volunteers (9 male, 4 female) with ages of 29.3 ± 12.3 years (range, 12–50 years) in the Wood’s study, the normal R2* was 39.9 ± 2.8 s^−1^ (HIC = 1.17 mg/g dry weight)^6^.

In the presented series, there was no correlation between R2* and liver stiffness, neither for women nor for men. This is consistent with the results of other authors^42^.

Our study showed a clear difference in the mean liver R2* between men and women. Women had a statistically significantly lower R2*value (28.34 s^−1^) compared to men (29.57 s^−1^), *p* = 0.040. This was expected as premenopausal women lose iron due to menstruation. However, premenopausal women lose iron due to menstruation so our women-dominated population of young healthy volunteers should has a lower iron level than a more general population that includes more elderly people and men.

Also, in this study, we only used the R2* images on the scanner, generated by the manufacturer IDEAL-IQ complex-based fat-water R2* model. There are many other magnitude-based R2* models, for example, Yokoo et al.^43^ studied the effect of Rician and non-Rician noise on the accuracy of R2* estimation in 7 different magnitude-based models, and found, the agreement of the R2* estimation was excellent in patients with low ferritin but poor in patients with high ferritin, as expected. Tipirneni-Sajja et al.^44^ simulated 5 different magnitude-based R2* models (GRE-A, GRE-B, GRE-C, UTE-A, and UTE-A), and found, the constant offset monoexponential model overestimated the R2* at the lowest simulated SNR, and the monoexponential fitting with noise subtraction is the most robust and accurate R2* model for ultrashort TE (UTE) sequence.

A limitation of the presented work is the narrow age range of the studied group. Therefore, it was not possible to analyze the correlation of R2* with age. Further studies should include individuals within a wider age range to investigate such potential dependence. Equally interesting would be the analysis of LIC in menstruating and postmenopausal women.

The lack of liver biopsies as an alternative measure of iron content can also be considered a weakness of this study, but the design of the work a priori excluded such a reference point.

By measuring liver R2* during routine MR testing and referring it to the reference values, patients with mild elevation of ferritin blood levels and no iron overload can avoid invasive diagnostics (e.g., liver biopsy) or invasive blood-letting tests. It should be remembered that an increase in ferritin levels might occur in the course of other disease processes such as hepatitis, obesity, or alcohol consumption^45^.

Also, the MR method used in this study to assess the hepatic iron level has the additional advantage of being able to evaluate the total amount of iron in the whole liver, as opposed to the localized assessment from biopsy, which can cause a significant diagnostic error in the case of an inhomogeneous distribution of iron in the liver^46^.

**Figure 6:**
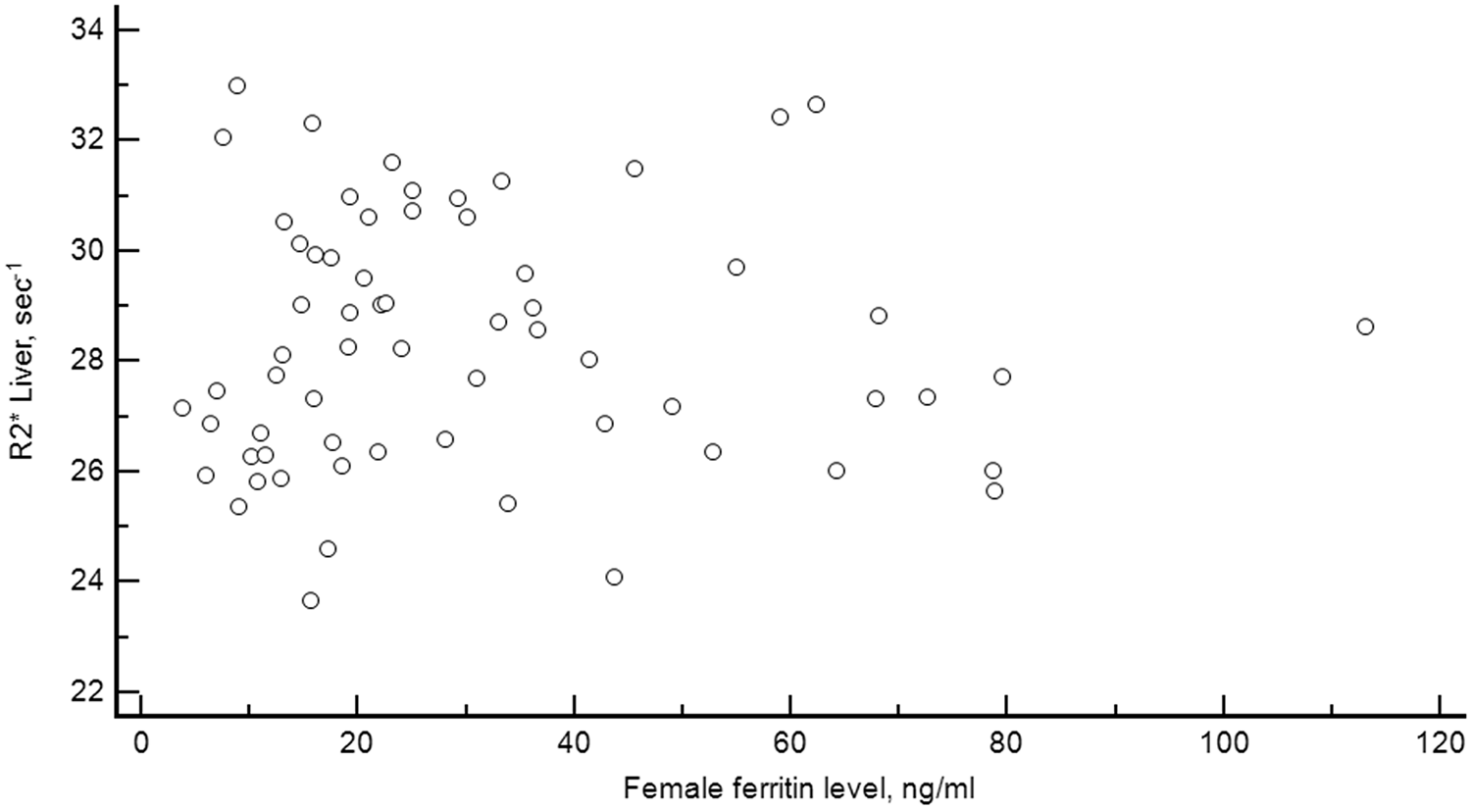
Relationship between ferritin level and liver R2* for females. Scatterplot diagram showing the relation between ferritin level and liver R2* in the 64 healthy female volunteers.

In summary, MRI R2* has been developed to accurately measure hepatic iron concentration. Our study has shown a normative hepatic R2* value of less than 38 s^−1^ in a women-dominated (64%) and relatively large population of healthy young people, which is a valuable reference for the medical community (Fig. 6).

## Data Availability

The data that support the findings of this study are available from the corresponding author upon reasonable request.
